# Molecular Detection of Streptococcus mutans and Streptococcus sobrinus in Dental Plaque Samples in Children Aged Six to Nine Years

**DOI:** 10.7759/cureus.32672

**Published:** 2022-12-18

**Authors:** Sunil Pandey, Rachana L Patnayak, Prafulla K Khodiar, Dnyanesh Amle, Anil Pandey, Pragya Tripathi

**Affiliations:** 1 Department of Pedodontics and Preventive Dentistry, Government Dental College, Raipur, IND; 2 Department of Microbiology, All India Institute of Medical Sciences Bibinagar, Bibinagar, IND; 3 Department of Biochemistry, Pt Jawaharlal Nehru Medical College, Raipur, IND; 4 Department of Biochemistry, All India Institute of Medical Sciences Nagpur, Nagpur, IND; 5 Department of Periodontology, Indraprastha Dental College and Hospital, Sahibabad, IND

**Keywords:** polymerase chain reaction, dental caries, dental plaque, streptococcus sobrinus, streptococcus mutans

## Abstract

Context

Dental caries is a widespread threat, usually in children, although it has been observed at other stages of life. Various pieces of literature have confirmed the prevalence of *Streptococcus mutans* and *Streptococcus sobrinus *in the progression of the disease. However, establishing procedures to detect these species remains a challenge, posing a barrier to treatment plans.

Aim

The aim of this study is to detect the species in dental plaque samples from children aged six to nine years by polymerase chain reaction (PCR) and correlate their prevalence in various dentitions.

Material and Methods

This is an observational analytical cross-sectional study conducted in a tertiary care dental hospital. After sample isolation, microbiological processing was performed, genomic DNA was isolated, and PCR run was performed using specific primers to detect the species. SPSS for Windows Version 17 (IBM Corp., Armonk, NY) and Microsoft Excel (Microsoft Corporation, Redmond, WA, USA) were used to perform statistical analysis. A p-value of <0.05 was considered statistically significant.

Results

The technique could identify *S. Mutans* and *S. Sobrinus* in a short turnaround time. The frequency of *S. mutans* and *S. sobrinus* infections was higher in individuals with dental caries.

Conclusions

Molecular detection via PCR is a reliable, economical, and less time-consuming method for detecting *S. mutans* and *S. sobrinus* in dental plaque samples.

## Introduction

Dental caries, also known as tooth decay or dental cavities, is the most widespread non-communicable chronic disease globally, affecting people throughout their lifetime and causing pain, discomfort, disfigurement, and even death; hence, it is a significant public health issue problem [1]. During the last few decades, many measures have been developed, tested, and implemented to combat this disease, and have benefitted many people worldwide. Regardless of the endeavors in this area, a large part of the global population still suffers from dental caries, which is a major cause of tooth loss. The Global Burden of Disease Study 2016 estimated that oral diseases affected at least 3.58 billion people worldwide, with caries of the permanent teeth being the most prevalent of all conditions assessed. Globally, it is estimated that 2.4 billion people suffer from caries of permanent teeth and 486 million children suffer from caries of primary teeth [2]. India has had only a single National Oral Health Survey conducted in 2002, which stated that the DMFT (decayed, missing, and permanent filled teeth) index score for Indian children was around 2, and caries prevalence was increasing with age from 51.9% to 63.1% in the 5- to 15-year age group, respectively [3].

According to the present data, the worldwide prevalence of dental caries has lessened with time. This rate differs significantly between developed and developing countries or middle- to low-income countries. The developed or high-income countries have shown better results, and thus a high rate of decline in dental caries can be seen. This decline can be ascribed to the use of fluoride as well as better and established preventive programs for the disease. In contrast, the reduction rate is less in middle- to low-income countries because of the increasing consumption of sugar and refined foods [4]. The disease, thus, is highly associated with socio-economic status, with high prevalence rates among poor and disadvantaged population groups.

Children in the mixed dentition stage are prone to poor oral hygiene due to carefree age, emotional stress, increased frequency in the intake of refined sugar, soft and sticky foods, shedding of deciduous, and the eruption of permanent teeth. This period is critical from the point of view of the normal development of occlusion and the preservation of first molars from dental caries [5]. Localized dissolution and destruction of the calcified tissues often result in cavitation as a result of the disease. It interferes with regular food intake, speech, self-esteem, and routine activities, affecting the overall health status of the children [6].

*Streptococcus mutans* (*S. mutans*) and *Streptococcus sobrinus* (*S. sobrinus*) are the most common etiological agents for dental caries [7]. It has been observed that *S. mutans* is more prevalent than S. sobrinus, with the latter being more closely associated with increased caries. Several methods are under practice in order to identify these organisms. Bacterial isolation followed by subsequent biochemical and immunologic tests have been used as a conventional method. DNA (deoxyribonucleic acid) probes and serological methods have improved their efficiency. However, due to their tediousness, poor sensitivity, and time-consuming limitations, polymerase chain reaction (PCR, is considered a better option because of its simple, rapid, and reliable identification of the species [7,8]. PCR is readily available and can be used to detect vast arrays of pathogens. The present study focused on dextranase genes (dex) of cariogenic species for detection and identification. Dextranase is an enzyme that hydrolyses glucans in a plaque matrix and is also believed to play a significant role in the pathogenesis of dental caries. The enzymes produced by *S. mutans* and *S. sobrinus* have been well studied. So far, the PCR primers have been designed for the *S. mutans* dex gene and the *S. sobrinus* dex gene to establish species-specific PCR methods for their detection and identification.

Age-specific data are essential to determine the most vulnerable group for the disease. Identification and determination of the prevalence of *S. mutans* and *S. sobrinus* are of primary importance in understanding the initiation and progression of this disease. Furthermore, their detection will provide better treatment and prevention strategies. Therefore, we aimed to detect *S. mutans* and *S. sobrinus* in dental plaque samples from children aged six to nine years by PCR and correlate their prevalence in various dentitions.

## Materials and methods

This observational, analytical cross-sectional study was carried out at the Department of Pedodontics and Preventive Dentistry, Government Dental College, Raipur, Chhattisgarh, India, in collaboration with the Center for Genetic Diseases and Molecular Biology, Department of Biochemistry, Pt. Jawaharlal Nehru Memorial Medical College, Raipur. The study enrolled six- to nine-year-old children of either gender presenting with dental caries. All individuals with symptoms suggestive of dental caries further confirmed by local examination and investigations were included in the study, while children presenting with obvious signs of malnutrition or with other serious illnesses or those whose parents were not willing to give consent were excluded. Ethical clearance was obtained from the Institutional Ethical Committee of the Government Dental College. The study was conducted in accordance with the guidelines of the Declaration of Helsinki.

After obtaining informed written consent, a thorough dental examination of each child was carried out, with radiological investigations wherever necessary and findings noted. The presence of deciduous teeth, permanent teeth, decayed teeth, and missing teeth were all noted. After confirming dental caries, dental plaques were removed by cleaning teeth, and 1-15 mL of dental wash containing plaque was obtained afresh. The sample was used to isolate strains of *S. mutans* and *S. sobrinus*, as described in detail further.

Microbiological processing

The plaque samples were vortexed and plated on mitis salivarius bacitracin (MSB) agar medium. A loopful of inoculums was obtained from the sample and streaked on freshly prepared plates. The plates were incubated anaerobically at 37°C for 48 hours, and the colonies were identified using colony morphology. The typical colonies for each sample plate were transferred to Brain Heart infusion (BHI) broth (HiMedia, Maharashtra, India). The broth was incubated at 37°C for 18 hours.

Chromosomal DNA extraction

The BHI broth was first measured for its absorbance at 600 nm to test its logarithmic phase. After obtaining adequate absorption, the samples were processed for DNA isolation using the phenol-chloroform method. For this, 2 mL of broth was centrifuged briefly to obtain the cell pellet, followed by its resuspension in Tris-EDTA (TE) buffer. To it, 10% sodium dodecyl sulfate (SDS) and 5 µL of 20 mg/mL proteinase K were added and incubated for one hour at 37°C to ensure cell lysis. Furthermore, freshly prepared phenol-chloroform was added and centrifuged. The aqueous phase was separated, and DNA was precipitated by adding sodium acetate and chilled absolute ethanol and washed with 70% ethanol. To the DNA present as a white pellet, TE buffer was added and stored at 4°C. Qualitative estimation of DNA was performed by agarose gel electrophoresis using 2% agarose gel and visualized in a UV transilluminator. DNA quantification was done through UV spectroscopy by preparing 1:100 dilutions of the extracted DNA in distilled water, and the absorbance was read at 260 nm and 280 nm.

Polymerase chain reaction

PCR detection of the two species was performed using oligonucleotide primers designed to amplify 1272- and 1610-bp fragments of the dextranase genes of *S. mutans *and *S. sobrinus*, respectively. The SD1 and SD2 primers were used to specifically amplify a 1272-bp fragment in *S. mutans*. The sequence of SD1 and SD2 were 5’-TAT GCT ATT GGA GGT TC-3’ (positions 973 to 992) and 5’-AAG GTT GAG CAATTG AAT CG-3’ (positions 2225 to 2244), respectively. SOF14 and SOR1623 primers were used to specifically amplify a 1610-bp fragment in *S. sobrinus*. The nucleotide sequence of SOF14 and SOR1623 were 5’-TGC TAT CTT TCC CTA GCA TG-3’ (position 134-153) and 5’-GGT ATT CGG TTT GAC TGC-3’ (positions 1743-1726), respectively. The reaction was run for 30 cycles at 55°C annealing temperature, and the PCR products were subjected to electrophoresis on a 1% agarose gel and stained with ethidium bromide.

Statistical analysis

Data were expressed as percentage and mean ± SD or as median (range). A Kolmogorov-Smirnov analysis was performed to check the linearity of the data. The Student t-test was used to check the significance of the difference between two parameters in parametric data, and the Mann-Whitney U test was used to check the significance of the difference between two parameters in non-parametric data. Fischer’s exact test, or chi-square test, was used to analyze the significance of the difference between the frequency distribution of the data. A p-value of <0.05 was considered statistically significant. SPSS for Windows Version 17 (IBM Corp., Armonk, NY) and Microsoft Excel 2007 (Microsoft Corporation, Redmond, WA, USA) were used to perform the statistical analysis.

## Results

**Figure 1:**
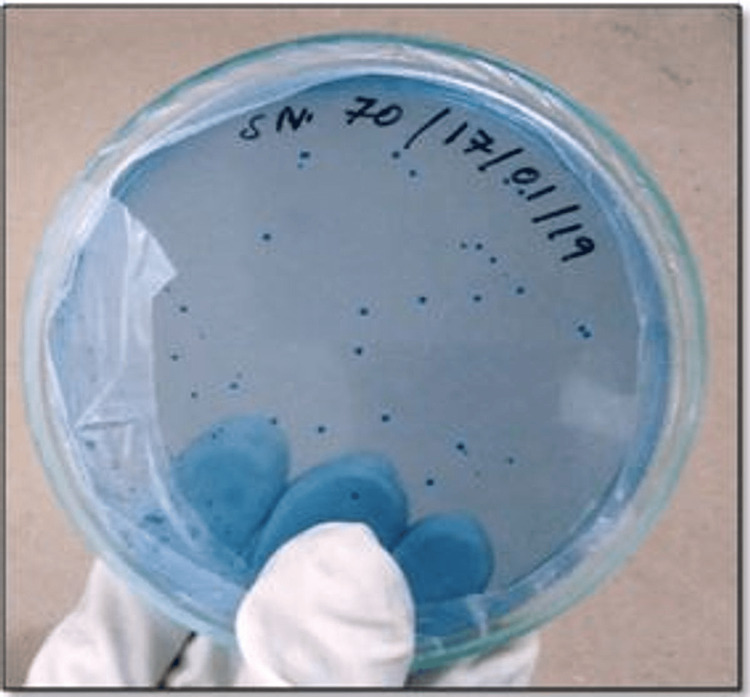
MS culture in mitis salivarius bacitracin media MS, mutans streptococci

The culture results obtained in MSB agar are shown in Figure 1.

**Figure 2:**
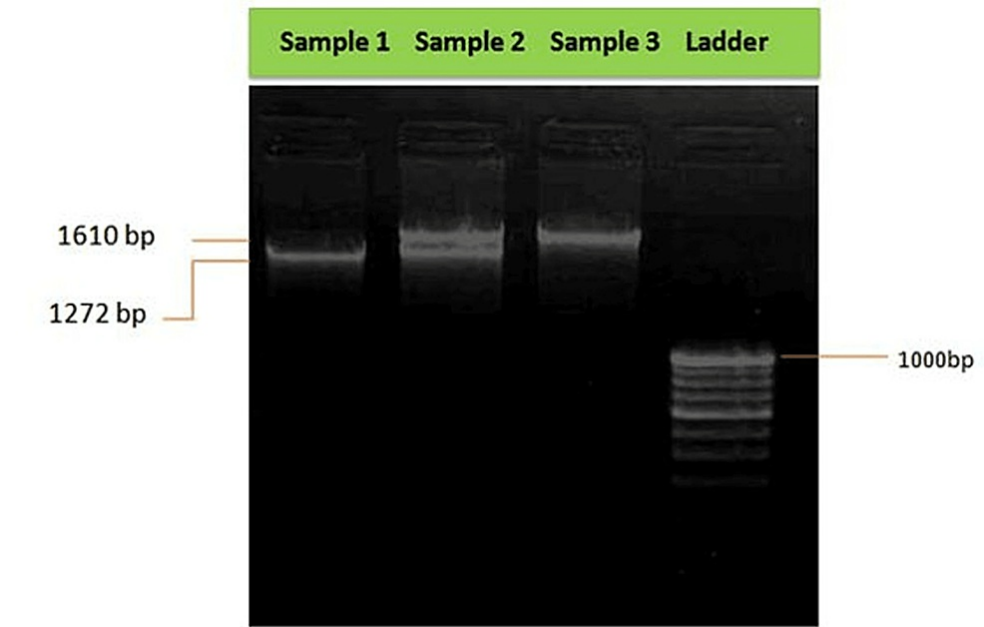
Findings of electrophoresis run for PCR products. A 100-bp ladder DNA was used, showing the last band of 1,000 bp; lane 1: 1272-bp band showing *S. mutans*; lane 2: both the 1272-bp band and the 1610-bp band show the presence of *S. mutans* and *S. sobrinus*; lane 3: 1610-bp band showing *S. sobrinus*. bp, base pairs; PCR, polymerase chain reaction

Figure 2 shows findings of electrophoresis run for PCR product. The 1272-bp band representing *S. mutans* (sample 1), both the 1272-bp band and the 1610-bp band representing the presence of *S. mutans* and *S. sobrinus* (sample 2), and the 1610-bp band representing *S. sobrinus* can be observed (sample 3).

Out of 100 individuals enrolled in the study, 31 (31%) were eight years old and 28 (28%) were seven years old (Table 1). This was followed by 23 (23%) individuals aged six years and 18 (18%) individuals aged nine years. Of the participants, 59 (59%) were males, while 41 (41%) were females. A maximum number of individuals had total teeth in the range of 26 to 30, followed by 29 (29%) individuals having total teeth in the range of 16 to 20. In addition, 51 (51%) individuals were observed to have deciduous teeth in the range of 16-20, followed by 11-15 in 40 (40%); 46 (46%) individuals were noted with ≤5 permanent teeth.

**Table 1:** Baseline characteristics of study subjects.

General characteristics		Frequency
Age (years)	6	23 (23%)
	7	28 (28.0)
	8	31 (31.0)
	9	18 (18.0)
Sex	Female	41 (41.0)
	Male	59 (59.0)
Total teeth	6–10	1 (1.0)
	16–20	29 (29.0)
	21–25	68 (68.0)
	26–30	2 (2.0)
No. of deciduous teeth	6–10	9 (9.0)
	11–15	40 (40.0)
	16–20	51 (51.0)
No. of permanent teeth	5	46 (46.0)
	6–10	31 (31.0)
	11–15	20 (20.0)
	16–20	3 (3.0)
Decayed deciduous teeth	5	91 (91.0)
	6–10	8 (8.0)
	11–15	1 (1.0)
Decayed permanent teeth	0	93 (93.0)
	1	3 (3.0)
	2	3 (3.0)
	3	1 (1.0)
Total decayed	5	91 (91.0)
	>5	9 (9.0)
Grossly decayed	5	98 (98.0)
	>5	2 (2.0)
Root stumps	2	98 (98.0)
	>2	2 (2.0)
Chief complaints	Visible caries	58 (58.0)
	Bad breath	55 (55.0)

Table 2 shows descriptive statistics for the individuals in the study. It suggested that the mean age group of the studied individuals was 7.44 ± 1.04. In the age group of six to nine years, the mean total teeth observed were 22.07± 2.37. The mean deciduous and permanent teeth were 15.48± 3.76 and 6.59± 4.50, respectively. Missing teeth in the studied individuals were noted to be 0.12 ± 0.57, while 2.56 ± 2.12 was the mean of total decayed teeth in individuals. Decayed deciduous and decayed permanent teeth were found to be 2.44 ± 2.11 and 0.12 ± 0.48, respectively. The mean of grossly decayed and root stumps was 0.84 ± 1.36 and 0.24 ± 0.71, respectively.

**Table 2:** Descriptive statistics of various parameters

Characteristics	Mean ± SD
Age (years)	7.44 ± 1.04
Total teeth	22.07 ± 2.37
No. of deciduous teeth	15.48 ± 3.76
No. of permanent teeth	6.59 ± 4.5
Missing teeth	0.12 ± 0.57
Total decayed teeth	2.56 ± 2.12
Decayed deciduous teeth	2.44 ± 2.11
Decayed permanent teeth	0.12 ± 0.48
Grossly decayed	0.84 ± 1.36
Root stumps	0.24 ± 0.71

Table 3 shows caries prevalence in schoolchildren with *S. mutans* alone and in combination with S. sobrinus. In the individuals, *S. mutans* and *S. sobrinus* were present in 68% and 67% of individuals alone, respectively, whereas 58% of individuals were positive for both the species, and in 23% of individuals, none were found. DMFT scores in individuals with different characteristics were compared using the Kruskal-Wallis test. All the characteristics matched for the DMFT score (p = 1.000). The dmft (decayed, missing, and filled primary teeth) and dmft+DMFT scores in individuals negative for both *S. mutans* and *S. sobrinus* were significantly lower than those positive for *S. mutans* alone (p<0.05). Similar observations were made when individuals without *S. mutans* and *S. sobrinus* were compared individually with those with *S. sobrinus* alone and with those positive for both species.

**Table 3:** Caries experience in school children with Streptococcus mutans alone and in combination with Streptococcus sobrinus *p<0.05 dmft, decayed, missing, and filled primary teeth; DMFT, decayed, missing, and filled permanent teeth

S. mutans	S. sobrinus	Number (%) of individuals	dmft*	DMFT	dmft+DMFT*
+	+	58 (58.0%)	3.17 ± 2.06	0.17 ± 0.58	3.35 ± 1.90
-	+	67 (67.0%)	3.26 ± 2.03	0.17 ± 0.58	3.43 ± 1.85
+	-	68 (68.0%)	3.13 ± 3.13	0.13 ± 0.46	3.26 ± 1.91
-	-	23 (23.0%)	2.22 ± 1.54	0.22 ± 0.74	2.43 ± 1.59

The distribution of bacteria among individuals with dental caries and without dental caries was performed, as shown in Table 4. In all individuals, those with both *S. mutans* and *S. sobrinus* were found to have dental caries, while those with *S. sobrinus* alone, *S. mutans* alone, or the absence of both did not have dental caries.

**Table 4:** Distribution of bacteria among subjects with visible caries and without visible caries

Microorganisms present		Visible caries		Total
*S. mutans*	*S. sobrinus*	Present	Absent	
+	-	0 (0)	10 (100)	10
-	+	0 (0)	9 (100)	9
+	+	58 (100)	0 (0)	58
-	-	0 (0)	23 (100)	23
Total		58 (58)	42 (42)	100

Bacteria among individuals with dental caries and the caries-free group were compared, as shown in Table 5. A significant difference was observed between dental caries group and the dental caries-free group for the various combinations of *S. mutans* and *S. sobrinus* (p<0.0001).

**Table 5:** Comparison of bacteria among individuals with visible caries and visible caries-free group

Variable		Visible caries group (n = 58)	Visible caries-free group (n = 42)	p-Value	
*S. mutans*	Present	58 (100)	0 (0)	<0.0001	
					Absent	0 (0)	32 (76.19)	
	*S. sobrinus*	Present	58 (100)					9 (21.43)	<0.0001	
					Absent	0 (0)	33 (78.57)			
*S. mutans *and* S. sobrinus*		Present	58 (100)	0 (0)				<0.0001		
					Absent	0 (0)	23 (54.76)			

A comparison of various parameters between *S. mutans *and *S. sobrinus* was assessed using the chi-square test, as shown in Table 6. Gender in the studied individuals was found to be matched for *S. mutans* and *S. sobrinus* (p = 0.40). Dental caries was significantly higher in *S. mutans* and *S. sobrinus* positive cases in comparison to those absent for the respective organisms (p <0.0001).

**Table 6:** Comparison of various parameters between S. mutans and S. sobrinus

Characteristics				Chi-square	P-value
		*S. mutans* (N=68)	*S. sobrinus* (N=67)		
Gender	Female	23	25	0.18	0.40
		33.8%	37.3%		
	Male	45	42		
		66.2%	62.7%		
Age (years)	6	15	17	0.25	0.97
		22.1%	25.4%		
	7	24	22		
		35.3%	32.8%		
	8	18	18		
		26.5%	26.9%		
	9	11	10		
		16.2%	14.9%		
Decayed		55	55		
		80.9%	80.9%		
Grossly decayed		24	25	0.03	0.5
		35.3%	37.3%		
Visible caries		58	58	-	1
		85.3%	86.6%		

Individuals were compared according to infection by *S. mutans* and *S. sobrinus* and caries prevalence, as shown in Table 7. All the individuals with *S. mutans* alone or with both *S. mutans* and *S. sobrinus* had a 100% prevalence of dental caries. In contrast, those with *S. sobrinus* alone had an 86.57% prevalence of dental caries. However, dental caries was not prevalent in those negative for *S. mutans* and *S. sobrinus*.

**Table 7:** Comparison between subjects according to infection by S. mutans and/or S. sobrinus and caries prevalence

Species	Visible caries group, N (% of total)	Visible caries-free group, N (% of total)	Total	Visible caries prevalence (%)
*S. mutans* +	58 (100)	0 (0)	58	100
*S. sobrinus* +	58 (100)	9 (21.43)	67	86.57
*S mutans* and *S. sobrinus* +	58 (100)	0 (0)	58	100
*S. mutans* and *S. sobrinus* negative	0 (0)	23 (54.67)	23	0

## Discussion

Dental caries is one of the most common oral problems affecting children globally. It can be seen in children of all ages, involving deciduous and permanent teeth. Dental caries is an infectious disease and a major public health problem widespread worldwide. Members of the *Mutans streptococci* (MS) - *S. mutans* and *S. sobrinus* - have been implicated as the major etiological agents in this disease and are the most commonly found MS species in humans [7]. The correct identification and differentiation of these species are necessary to understand the early phases of colonization of the oral cavity and, thus, design appropriate treatment methods.

Many studies have been conducted to study the prevalence of dental caries in different parts of India. However, despite the importance of epidemiological studies in school-going children, limited research is carried out. In the present study, 100 children between six and nine years of age were enrolled. Among different sampling methodologies, a plaque sample was preferred over saliva as detection levels of MS species were stated to be higher in plaque [9]. The plaque samples were isolated and subjected to molecular study to establish a direct and more reliable method of identifying these cariogenic species.

The method of identification of MS species was precise as species-specific primers were used to carry out PCR. Oligonucleotide primers were obtained for the dex gene of *S. mutans* and *S. sobrinus*. These primers were expected to amplify 1,272 bp and 1,610 bp in *S. mutans* and *S. sobrinus* species. Figure 2 shows the PCR results obtained on viewing under gel doc. Each primer pair gave a single amplicon specifically. PCR detection of cariogenic bacteria in the study individuals was observed and interpreted. The presence of *S. mutans* and *S. sobrinus* in children aged six to nine years in this study was found to be 68% and 67%, respectively, in agreement with the results of Franco et al., Salman et al., Okada et al., Amoroso et al., and Klein et al. [6,8-11]. However, this result was not in accordance with Japanese individuals aged 6-30 years, as reported in a study conducted by Oda et al., where the prevalence of *S. sobrinus* was higher than S. mutans. Both *S. mutans* and *S. sobrinus* were found positive in 58 (58%) individuals, in accordance with Oda et al.’s study [12]. Similarly, Okada et al. reported the prevalence to be 53.9% [6]. The small difference can be attributed to the fact that the number of individuals differed in the two studies. While Franco et al. reported zero prevalence of both the species residing together in study individuals [6].

Both *S. mutans* and *S. sobrinus* were found to be negative in 24 (36.92%) individuals. The absence of bacteria from the sample collection sites might be the reason for not detecting these bacteria. The number of *S. mutans* and *S. sobrinus* was below the detection limits in plaque samples, indicating that the involvement of nonspecific bacteria in caries formation supports the nonspecific plaque hypothesis. A similar observation was made by Salman et al. [8].

Significant male preponderance was noted in the frequency of *S. mutans*. However, in a previous study, no significant difference was noted in males and females in terms of the presence of S. mutans [13,14]. Also, the frequency of *S. mutans* infection was significantly higher in individuals with dental caries, similar to the study conducted by Franco et al. [6]. The association of various parameters with *S. sobrinus* was performed using the chi-square test. The frequency of *S. sobrinus* infection was significantly higher in individuals with dental caries (p=<0.001), which was similar to the study done by Soni and Vasavda [14].

The dmft score was found to be higher in individuals with *S. sobrinus* positive and *S. mutans* positive compared to both positive; also, dmft score was higher in both positive (S+M) individuals compared to both negative individuals. But these differences failed to reach statistical significance. The dmft+DMFT score in individuals with different characteristics was compared using ANOVA. All the characteristics were found to be matched for dmft+DMFT score in individuals (p = 0.076). DMFT scores in individuals with different characteristics were also compared, and the groups were found to be matched for DMFT scores (p = 1.000).

## Conclusions

In conclusion, the present cross-sectional study results indicate that individuals with *S. mutans* and *S. sobrinus* presented dental caries prevalence. Also, PCR showed a strong distinguishing ability for differentiation between these two species, suggesting that it is an ideal technique suitable for epidemiological studies. The conventional procedures for the detection of cariogenic species usually take a long time (~one week). Molecular method of detection such as PCR as described previously shortens the time and thus is an ideal technique suitable for epidemiological studies.
